# Editorial: Epilepsy syndromes: pathophysiology and managements

**DOI:** 10.3389/fneur.2023.1325553

**Published:** 2023-11-13

**Authors:** Hua-Jun Feng, Tao Sun, Bo Xiao

**Affiliations:** ^1^Department of Anesthesia, Critical Care and Pain Medicine, Massachusetts General Hospital, Boston, MA, United States; ^2^Department of Anesthesia, Harvard Medical School, Boston, MA, United States; ^3^Department of Neurosurgery, General Hospital of Ningxia Medical University, Yinchuan, China; ^4^Ningxia Key Laboratory of Cerebrocranial Disease, Ningxia Medical University, Yinchuan, China; ^5^Department of Neurology, Xiangya Hospital, Central South University, Changsha, China; ^6^National Clinical Research Center for Geriatric Disorders, Xiangya Hospital, Central South University, Changsha, China; ^7^Clinical Research Center for Epileptic Disease of Hunan Province, Central South University, Changsha, China

**Keywords:** epilepsies, electroclinical features, genetic mutations, heterogeneity, pathophysiological mechanisms, treatments

An epilepsy syndrome is defined as “a characteristic cluster of clinical and electroencephalogram (EEG) features, often supported by specific etiological findings (structural, genetic, metabolic, immune, and infectious)” (1). An epilepsy syndrome is usually age dependent and often comorbid with cognitive impairment and psychiatric abnormality (1). The types of epilepsy in epilepsy syndromes include focal, generalized, or focal and generalized onset epilepsy (1, 2). Epilepsy syndromes with developmental and epileptic encephalopathy (DEE) describe a group of syndromes that are associated with developmental deficits, likely resulting from both developmental etiology and seizure activity (1). These drug-resistant epilepsy syndromes are usually due to genetic mutations, characterized by early onset, abnormal EEG, and high risk of sudden unexpected death in epilepsy (SUDEP) (3). The pathophysiological mechanisms underlying epilepsy syndromes are incompletely understood, and the clinical management is often unsatisfactory. This Research Topic collected articles on epilepsy syndromes with DEE and those with a broader definition.

Infantile epileptic spasm syndrome (IESS), featured by epileptic spasms, usually afflicts children under two. It is called West syndrome when IESS is combined with a hypsarrhythmia on EEG and developmental delay (4). Arai et al. performed a retrospective study to analyze the factors that predict the long-term prognosis of seizures and their development in patients with IESS. They found that several factors, e.g., the persistence of epileptic spasms or tonic seizures after 90 days of onset, are associated with the poor outcomes of IESS 3 years after the epileptic spasm onset. These findings help guide the selection of appropriate and timely interventions. Although an established treatment regimen is unavailable for IESS, several medications, including vigabatrin, are considered the first-line treatment (4). Kim et al. utilized an EEG functional connectivity analysis to predict the clinical responses to vigabatrin-based treatment of IESS. They observed that the positive responders to vigabatrin-based treatment exhibited higher beta connectivity in the parieto-occipital areas. In contrast, the poor responders displayed a lower alpha connectivity in the frontal regions. Their data also indicated that the patients with fewer abnormal brain structural imaging findings were likely positive responders. This study provides an approach to evaluating the effect of a treatment on IESS using EEG analysis.

Dravet syndrome is probably one of the best-known epilepsy syndromes with DEE. Dravet syndrome exhibits significant electroclinical and genetic heterogeneity even though it is mainly caused by mutations in *SCN1A*, the gene for the α subunit of the voltage-gated sodium channel (5). Tahara et al. investigated the developmental changes in brain excitability in *Scn1a* knockout rats. They found that neural activity increased at a particular developmental stage in heterozygous knockout rats, which may be attributed to immature GABAergic neurotransmission. The phenotype of the heterozygous knockout rats was mild, with rare spontaneous seizures and no SUDEP during the experimental period, in general agreement with another study of *Scn1a* knockout rats (6). Interestingly, depending on the genetic background, the *Scn1a* knockout mice exhibited diverse phenotypes, ranging from no overt spontaneous seizures to frequent spontaneous seizures with a high SUDEP rate (7). Li et al. reported an *SCN1A* gene missense variant in a family with genetic epilepsy with febrile seizures plus (GEFSP). The heterozygous variant c.5725A>G (p.T1909A) was proposed as the genetic cause of the GEFSP phenotype in the family. While the initial seizures were provoked by fever in both patients, febrile seizures developed into afebrile seizures in one patient, and no afebrile seizures occurred in the other, showing the phenotypical heterogeneity with the same genetic basis of GEFSP.

Accumulating evidence indicates that the cerebellum is involved in epilepsy. Wang et al. conducted a clinical study to examine the microstructural changes in the cerebellum and cerebellar–cerebral functional connectivity in patients with temporal lobe epilepsy (TLE), with subgroup analysis including the cases with/without hippocampal sclerosis (HS)/focal-to-bilateral tonic–clonic seizures (FBTCS) and those with different lateralization. Extensive gray matter atrophy was observed in the cerebellum, and the cerebellar microstructural alterations were mainly found in patients with HS and FBTCS. Both reduced and increased cerebellar–cerebral functional connectivity were observed in patients with TLE, with decreased cerebellar–cerebral functional connectivity associated with the impairment of cognitive function.

Lennox–Gastaut syndrome (LGS) is an epilepsy syndrome with DEE (3). Liu et al. evaluated the outcomes of the surgical treatment of LGS due to viral encephalitis. The surgical strategies were determined mainly by neuroimaging findings as LGS caused by viral encephalitis exhibits considerable structural brain lesions. If magnetic resonance imaging (MRI) displays apparent bilateral lesions, palliative surgery such as corpus callosotomy can substantially reduce seizures. Alternatively, curative surgery becomes a challenging option if an MRI shows apparent unilateral lesions. In this case, analyzing the MRI findings in both the residual and acute phases is important. In addition, positron emission tomography (PET) images can be used for verification. Consistent findings in both MRI and PET indicate a better surgical outcome.

Nitrogen permease regulator-like 3 (NPRL3) is a subunit of GATOR1, which inhibits mTORC1 to suppress mTOR signaling (8). *NPRL3* gene mutations can lead to *NPRL3*-related epilepsy (NRE). Zhang et al. genetically identified 11 cases of NRE and performed clinical and genetic analysis by combining these cases with those reported up to date in the literature. NRE displays a significant clinical heterogeneity; the most common epilepsy types are sleep-related hypermotor epilepsy, frontal lobe epilepsy, and TLE. The authors also reported infantile spasms as a new phenotype. NRE also exhibits a high genetic heterogeneity; the most common *NPRL3* gene mutations are c.275G>A, c.745G>A, and c.1270C>T. Analysis of the treatment indicates that sodium channel blockers, including oxcarbazepine, are effective for NRE.

Many patients still use traditional antiseizure medications (ASMs) as a first-line treatment for controlling seizures. Hersi et al. studied the clinical factors that influence the doses of ASMs in patients with newly diagnosed epilepsy. Their data showed that the most prescribed ASMs are oxcarbazepine, followed by valproic acid, carbamazepine, and lamotrigine, all of which are sodium channel blockers. The seizure-free doses of oxcarbazepine are mainly influenced by the ages of the patients, with a lower dose in older patients. The seizure-free doses of valproic acid are instead sex dependent, with a higher dose in male patients.

## Author contributions

H-JF: Writing—original draft, Writing—review & editing. TS: Writing—review & editing. BX: Writing—review & editing.
